# Remanufacturing System with Chatter Suppression for CNC Turning

**DOI:** 10.3390/s20185070

**Published:** 2020-09-07

**Authors:** Karol Miądlicki, Marcin Jasiewicz, Marcin Gołaszewski, Marcin Królikowski, Bartosz Powałka

**Affiliations:** Department of Mechanical Engineering and Mechatronics, West Pomeranian University of Technology, Szczecin, al. Piastów 19, 70-310 Szczecin, Poland; marcin.jasiewicz@zut.edu.pl (M.J.); gm37274@zut.edu.pl (M.G.); marcin.krolikowski@zut.edu.pl (M.K.); bartosz.powalka@zut.edu.pl (B.P.)

**Keywords:** feature recognition, geometry recognition, 3D scanning, chatter, machining assistance, machining stability, receptance coupling, finite element model, refactoring

## Abstract

The paper presents the concept of a support system for the manufacture of machine spare parts. The operation of the system is based on a reverse engineering module enabling feature recognition based on a 3D parts scan. Then, a CAD geometrical model is generated, on the basis of which a machining strategy using the CAM system is developed. In parallel, based on the geometric model, a finite element model is built, which facilitates defining technological parameters, allowing one to minimize the risk of vibrations during machining. These parameters constitute input information to the CAM module. The operation of the described system is presented on the example of machining parts of the shaft class. The result is a replacement part, the accuracy of which was compared by means of the iterative closest point algorithm obtaining the RMSE at the level of scanner accuracy.

## 1. Introduction

In the modern industry, in line with the latest trends and the idea of Industry 4.0, the objective is to increase automation and autonomy of production. The aim of these activities is to reduce the dependence of production plants on qualified machine operators and technologists, and to increase production efficiency. Therefore, the importance of intelligent production support systems and operators is growing. New machines are increasingly being equipped with support systems that significantly simplify operation and allow one to avoid costly errors, which are often due to a lack of operator experience. This approach reduces training costs, among other things, and makes the plant independent of qualified personnel. Owing to this approach, it is possible to assign employees to operate the machine, even if they do not have specialist knowledge of how to produce workpieces. It may turn out to be particularly important in the maintenance departments of industrial companies or in onboard ships applications, in the engine department. An integral part of the tasks performed by these units is the damaged parts replacement. If the spare part is available, the repair can be performed instantly. Otherwise, it is necessary to manufacture the parts by carrying out machining. It should be done as quickly as possible; therefore, any mistakes made or unforeseen difficulties such as vibrations during machining are unacceptable. In such situations, the assistance systems can be invaluably helpful.

Available tools’ supporting production can be divided into two groups. The first group are tools used mainly by managers, executives, and technologists. The available production support tools include production management systems, production planning tools, parts design support systems, production quality control systems and reverse engineering tools, supporting, for example, CAD model building. These tools usually include advanced software installed on dedicated PCs. The second group is software and HMI interfaces dedicated to machine and equipment operators. Most often, they are integrated directly with machines. To this group we may include overlays on user interfaces or support systems based on virtual and augmented reality.

The machinery/machining industry in particular requires qualified staff, both at the stages of geometry design and development of the parts manufacturing process (technologists) and at the time of manufacture (machine operators). Therefore, tools for designers and technologists have developed over the years, along with the development of computer aided design and manufacturing techniques for machine parts. These tools include CAD systems to support the design of parts, CAM systems to facilitate the generation of machining programs and reverse engineering tools used, for example, to identify structural features. Most of these systems are integrated in commercial programs such as Dassault Systèmes SOLIDWORKS, Dassault Systèmes Catia, Siemens NX or PTC Creo. Reverse engineering systems are particularly advanced. Some commercial programs such as Ansys SpaceClaim have been equipped with a module supporting basic identification of structural features based on cross-sections in parallel planes. In parallel, newer and newer techniques are being developed to open up the geometry of parts [1], both in two and three dimensions [2]. V. B Sunil et al., in their work, present an intelligent system for recognizing prismatic part machining features from CAD models using an artificial neural network [3]. X. Lin et al. present a similar approach in [4], where a propose intelligent hybrid strategy is proposed for edge inconsistent feature detection by machine vision. The two deep neural networks are employed together in series, to first detect and then recognize polishing workpieces in an industrial environment; these were used by F. Liu et al. in [5].

Techniques are also being developed to identify structural features and dynamic properties for steel machine parts [6,7], as well as for modern composite parts [8,9].

In the machining industry, the key stage is to make a part from the blank. For this process, qualified CNC operators with extensive knowledge of G-code machining and programming technology are required, especially in view of the increasing demands concerning: machining time, surface quality [10], topographic control and continuous miniaturization, for which micrometers accuracy is required [11]. The machining of new polymer materials injected [12] or printed [13] is also a major challenge for operators. For many companies, the cost of hiring a qualified operator is too high and the time needed for training is too long. Therefore, the control systems of machine tools are extended with operator support systems, such as: compensation of temperature errors of ball screws [14] or 3D (three-dimensional) scanning vision system for positioning the workpiece [15]. More extensive CNC systems have additional built-in options, which include extensive graphical interfaces to facilitate the generation and analysis of G-code i.e., Siemens: Shopturn/ShopMill, Fanuc: Manual Guide. New graphic solutions with 3D elements, touch screens, remote controls and even gesture support are introduced to increase operator comfort. The latest systems also support users in the selection of technological parameters [16,17]. All systems mentioned above support an inexperienced operator in the machining process, owing to which he makes fewer mistakes and does not have to undergo expensive training.

However, despite the existence of systems to support designers/technologists and operators, so far, no solution has been developed to copy/manufacture a part without specialist knowledge including part design, development of machining technology, and manufacturing the part using a CNC machine. Currently, due to the development of the idea of Industry 4.0 and the Internet of Things, systems combining the tasks of a designer, a technologist and an operator are the object of increased research [18,19]. The popularity of these systems will continue to grow. This will be supported by the growing computing power of CNC systems and the increasing number of sensors integrated in machines.

This paper proposes an innovative system based on reverse engineering that allows for simple and intuitive copying of shaft type elements. The following procedures have been implemented in the system: scanning of parts, geometry reproduction, CAD model generation, simulation, selection of machining parameters (reduction of self-excited vibrations) and the generation of machining technology with the G-code. The main novelty presented in the article concerns the integration of known computational methods with the innovative feature recognition algorithm. The presented methodology may contribute to the development of manufacturing support systems. This can be particularly useful on ships, where access to qualified specialists and spare parts is significantly limited. Section 2 discusses and explains the various stages of system operation. Section 3 presents the results of the system operation and their discussion. Section 4 provides a summary and further plan for the development of the system.

## 2. CNC Machining Assistance System

The following subsections present the concept of a system in which a fully parametric CAD model is built with the use of reverse engineering, and then, on its basis, analyses supporting the technological process are conducted.

### 2.1. System Concept

**Figure 1:**
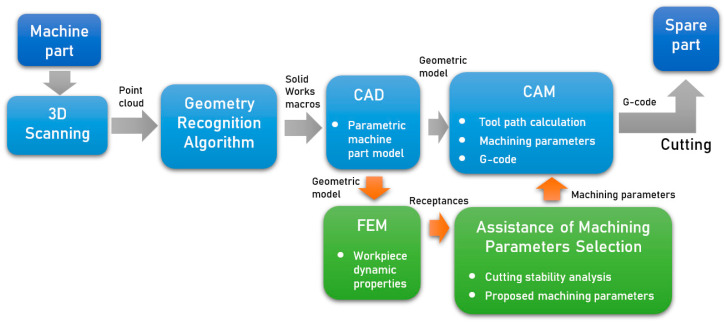
Block diagram showing the system concept.

The idea of operation of the developed system is based on the use of reverse engineering, in which a parametric CAD model is built on the basis of a 3D scan of the part. Then, the processing technology is developed on its basis, using the CAM module. One of the elements determining its effectiveness is the selection of appropriate technological parameters. In the presented system, this selection is supported by a module allowing one to minimize the risk of self-excited vibrations. This is possible on the basis of the analysis of dynamic properties of the workpiece. This analysis is carried out using the FEM model based on the CAD model. As a result of the proposed system, a part machining program is obtained with technological parameters that allow one to avoid vibrations during machining. The block diagram showing the system concept with the data flow is presented in Figure 1.

### 2.2. 3D Scanning

**Figure 2:**
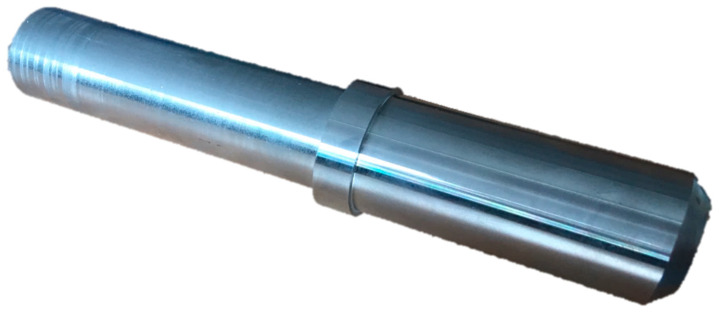
The part under consideration.

The part on the basis of which the operation of the system will be presented is a 183 mm long steel shaft with a maximum diameter of 37 mm made of steel, as shown in Figure 2. This is a part with a low degree of complexity, but due to the high average length/diameter ratio (L/D), there is a high risk of self-excited vibration during machining.

**Figure 3:**
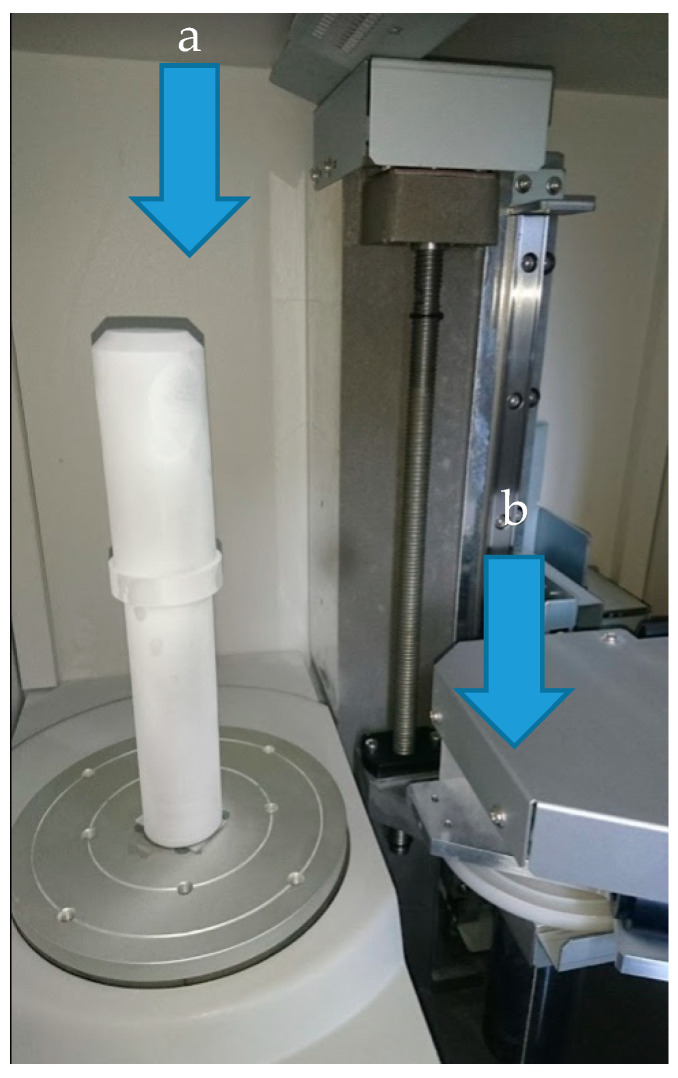
Experimental setup—shaft prepared for scanning; (**a**)—scanned object, (**b**)—scanning head.

Scanning was performed in rotary mode on the PICZA-LPX1200 scanner manufactured by Roland (1-6-4 Shinmiyakoda, Kita-ku, Hamamatsu-shi, Shizuoka-ken, 431-2103 Japan). After being covered with an anti-reflective white film, the object is placed in the axis of the rotary table, as shown in Figure 3. The following parameters were used during the scanning process: angular pitch—0.90 deg, lace cut in the axis of the object every 0.1 mm. XYZ point clouds with native size of 11.244 KB and 11.126KB (0.1 mm axial lace cut) were obtained.

### 2.3. Geometry Recognition Algorithm

**Figure 4:**
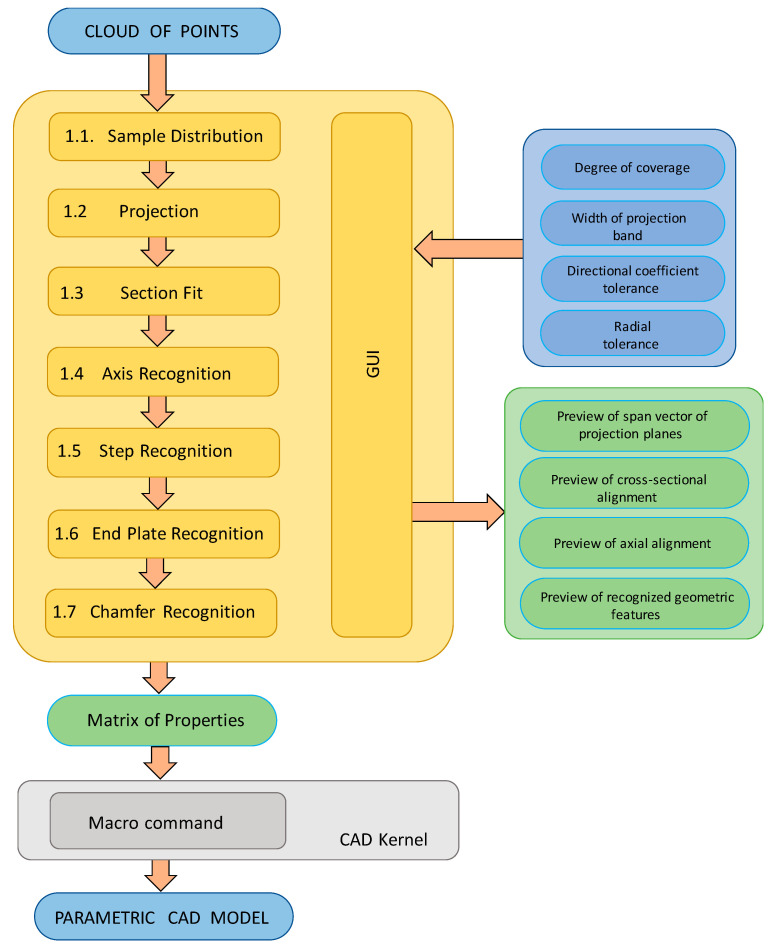
Diagram of cloud of points conversion to parametric geometry.

The next step was to convert the point cloud to parametric geometry. The implemented algorithms (Figure 4) identify the geometry of the shaft class parts based on cross-sections. The geometry of the identified part is then imported into SOLIDWORKS for further processing.

**Figure 5:**
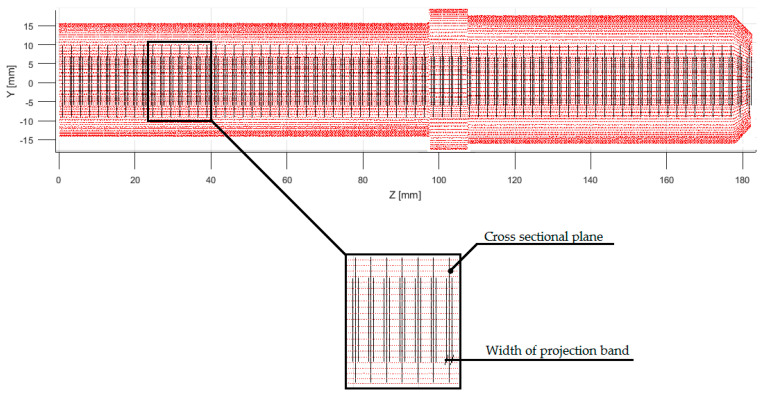
Span vector of cross-section planes with marked projection bands.

The operation of the geometry identification algorithm began with the determination of the vector of the span of planes (Figure 5), which was performed using the ‘Sample Distribution’ function. This function determines the vector based on the projection bandwidth and degree of coverage. The width of the projection band determines the symmetrical area around the section plane from which points from the cloud are projected. The degree of coverage is, on the other hand, a percentage parameter, which determines the total width of the projection bands. against the background of the *Z*-axis point cloud span (1).

(1) $$p=\frac{k\cdot s}{r}\cdot 100\%$$

where:

*p*—coverage parameter

*k*—number of scanning planes

*s*—width of a single projection band

*r*—span of the point cloud in the Z axis

Hence:

(2) $$k=\frac{p\cdot r}{s\cdot 100\%}$$

Then, on the basis of the designated span vector, using the ‘Projection’ function, points were projected on the cross-section planes. The circular cross-sections were adjusted using the ‘Section Fit’ function.

**Figure 6:**
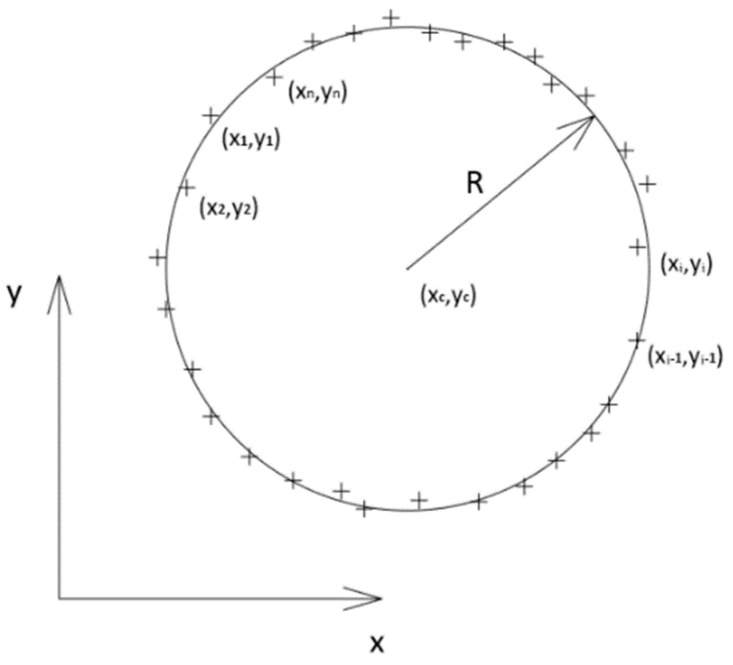
Approximation of the circular cross-section.

The ‘Section Fit’ function approximates a circular cross-section using the smallest squares method. To determine the coordinates of the center and the value of the section radius, a canonical equation of a circle (3) has been formulated, where $x_{i},y_{i}$ are the coordinates of the scanned i-th point, $x_{c},y_{c}$ are the coordinates of the center of the circle and R is the radius of the circle (Figure 6).

(3) $${\left(x_{i}-x_{c}\right)}^{2}+{\left(y_{i}-y_{c}\right)}^{2}=R^{2}$$

After the transformation of Equation (3), the general form of the circle equation is obtained:

(4) $$x_{i}^{2}+y_{i}^{2}=Ax_{i}+By_{i}+C$$

where: the constants *A*, *B*, *C* Equations (5)–(7) have been introduced to simplify the notation.

(5) $$A=2x_{c}$$

(6) $$B=2y_{c}$$

(7) $$C=-\left(A^{2}+B^{2}+R^{2}\right)$$

For each point (Figure 6) projected on the cross-sectional plane, the equation was formulated in the determined general form Equations (8)–(12).

(8) $$x_{1}^{2}+{y_{1}}^{2}=Ax_{1}+By_{1}+C$$

(9) $$x_{2}^{2}+{y_{2}}^{2}=Ax_{2}+By_{2}+C$$

(10) $$x_{i}^{2}+{y_{i}}^{2}=Ax_{i}+By_{i}+C$$

(11) $$x_{n-1}^{2}+y_{n-1}^{2}=Ax_{n-1}+By_{n-1}+C$$

(12) $$x_{n}^{2}+{y_{n}}^{2}=Ax_{n}+By_{n}+C$$

Next, the system of Equations (8)–(12) was transformed into a matrix notation Equation (13).

(13) $$\left[\begin{array}{ccc} x_{1} & y_{1} & 1\\ x_{2} & y_{2} & y\\ \ldots & \ldots & \ldots \\ x_{i} & y_{i} & 1\\ \ldots & \ldots & \ldots \\ x_{n} & y_{n} & 1 \end{array}\right]\left[\begin{array}{c} A\\ B\\ C \end{array}\right]=\left[\begin{array}{c} x_{1}^{2}+y_{1}^{2}\\ x_{2}^{2}+y_{2}^{2}\\ \ldots \\ x_{i}^{2}+x_{i}^{2}\\ \ldots \\ x_{n}^{2}+y_{n}^{2} \end{array}\right]$$

After solving the linear system of Equation (13), the values of constants A, B and C Equations (5)–(7) were obtained. Next, from their values, the coordinates of the circle center $x_{c},y_{c}$ Equations (14) and (15) and the radius R of the circle Equation (16) were determined.

(14) $$x_{c}=-\frac{A}{2}$$

(15) $$y_{c}=-\frac{B}{2}$$

(16) $$R=\sqrt{\frac{A^{2}+B^{2}+4C}{4}}$$

**Figure 7:**
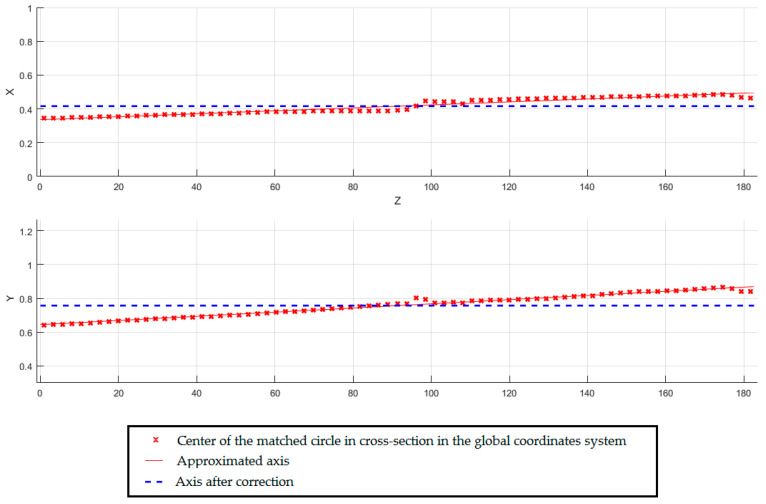
Overview of the shaft axis in XZ plane and YZ plane.

The circles were matched in the local coordinate system of the section plane. On the basis of the determined coordinates of centers using the ‘Axis Recognition’ function, the shaft axis in the global system was matched. Having regard to the defined directional coefficient tolerance, the matched axis has been corrected (Figure 7).

**Figure 8:**
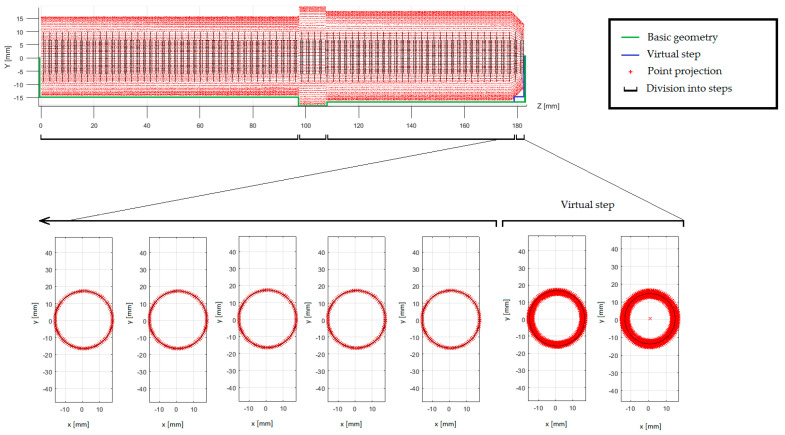
Overview of matched cross-sections with the apparent degree shown.

The identification of the shaft steps was made on the basis of the values of the cross-sectional radii. The radial tolerance parameter was the condition contained in the ‘Step Recognition’ module to recognize the belonging of successive cross sections to one shaft step. When i-th cross-section radius did not deviate within the radial tolerance from the cross-section radius i—1, both cross-sections were considered to belong to one shaft step. The detected sequence of less than three cross-sections is considered as an apparent degree, which is not taken into account in further proceedings (Figure 8). The appearance of the apparent degree results from the distortion of the radius by projecting points from the surfaces closing the shaft steps.

Using the ‘End Plate Recognition’ module, fragmented axial cross-sections were created in the transition areas of the shaft steps. The closing surfaces were adjusted using the ‘polyfit’ function from the Matlab library.

**Figure 9:**
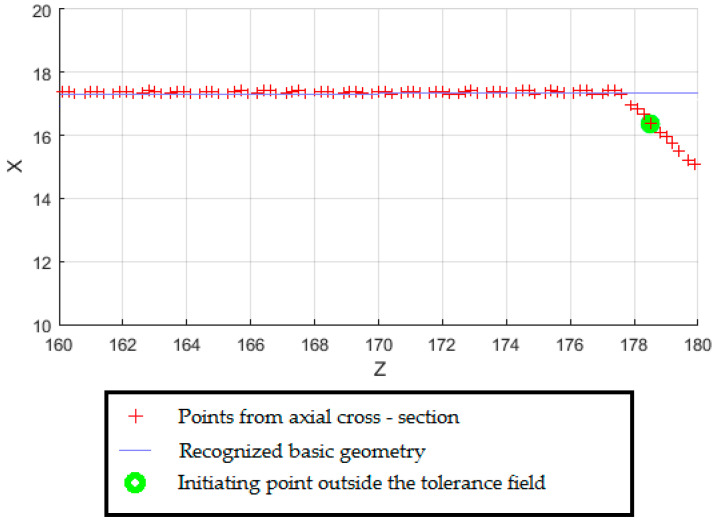
Chamfer detection based on axial cross-section.

In order to detect chamfering, fragmented axial cross-sections have been created in the end areas of the shaft steps. Using the ‘Chamfer Recognition’ module, a chamfer was determined by detecting the distance of projection points from the recognized basic geometry (Figure 9). The identified geometry with division into the base body and technological features has been recorded in the geometric properties matrix (Table 1).

**Figure 10:**
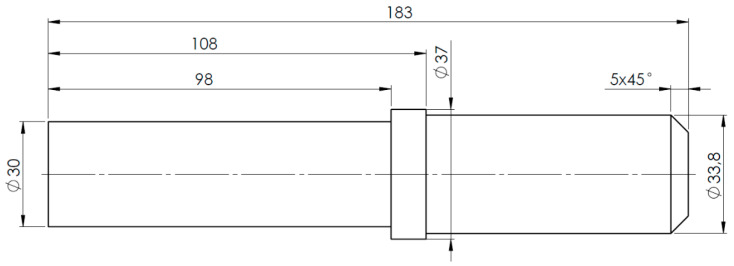
Identified part geometry.

The geometric properties matrix has been taken over by the macro command in Dassault Systèmes SOLIDWORKS 2018. The pseudo-code of the algorithm for importing geometry into SOLIDWORKS is shown in Table 2. First, the basic geometry was created by adding/extracting by rotation. Then, technological operations were added to the model. This approach allowed to reproduce the operations tree. The reconstructed geometry is shown in Figure 10.

### 2.4. Finite Element Model

Then, on the basis of the geometric model, a finite element model of the workpiece was built, in order to determine its characteristic frequency transition functions. The described frequency transition functions were the information necessary to determine the area of stability of machining using the CNC assistance module.

**Figure 11:**
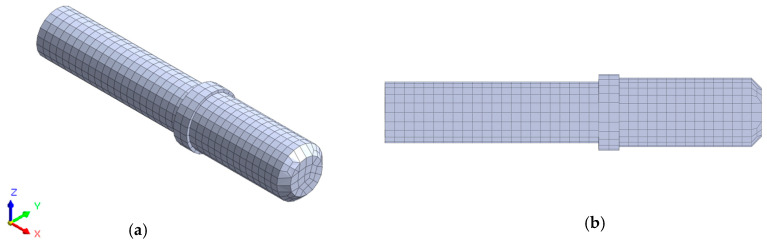
Discretized model of analyzed shaft, (**a**) isometric view and (**b**) cross-sectional view.

The finite element model is built using Midas NFX 2018 R1 software (Midas Information Technology Co. Ltd., Seongnam, Korea). In the first step, the geometric model was discretized using eight node, cubic, isoparametric finite elements (CHEXA) and six node, five-walled, isoparametric finite elements (CPENTA). The applied finite elements were characterized by linear shape functions and three translation degrees of freedom in each node. As a result of the discretization, a model consisting of 2.103 finite elements and 6.237 degrees of freedom was obtained. The discrete model is shown in Figure 11.

Due to the receptance method used in the CNC assistance module, on the basis of which the stable machining area is determined, an underdetermined model was adopted for further calculations.

**Figure 12:**
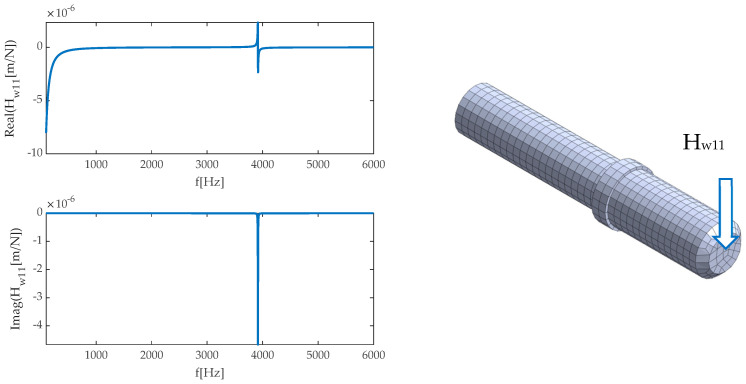
An example of FRF for the workpiece.

In the next step, using the Nastran Solver processor (SOL108), a set of receptance functions in the X and Z direction in the frequency ranging from 50 to 5000 Hz with a 1 Hz step was determined. Examples of calculation results as frequency response functions are presented in Figure 12.

### 2.5. Assistance of Machining Parameters Selection

**Figure 13:**
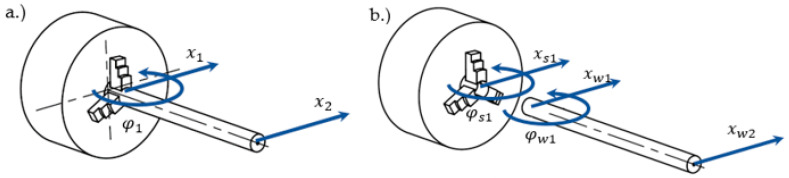
Receptance coupling system: (**a**) components, (**b**) coupled system.

In order to determine the process stability conditions for assessing the risk of self-excited vibrations, it is necessary to know the dynamic properties (as frequency response functions—FRFs) of the machine tool—workpiece system [20]. In the developed system, the system consists of a lathe spindle with a three-jaw chuck and a mounted workpiece. The dynamic properties of a given system can be determined with the application of modal synthesis, using the receptance coupling approach (RCA) [21,22]. This method allows the FRF function of a combined system to be determined (Figure 13a), having the dynamic properties of the components of which the system is composed (Figure 13b).

The dynamic properties of the machine tool are reduced to the properties of the spindle with the three-jaw chuck, and the experimental extended inverse receptance coupling (EIRC) method, described in detail in [23]. This method allows one to take into account the rotational degrees of freedom (RDOF) of the system, necessary to properly model the way the workpiece is clamped in the three-jaw chuck. Moreover, while modelling the properties of the spindle, it is possible to add an extra length of the machined part resulting from the use of a longer workpiece. The dynamic properties of the spindle remain unchanged over time; thus, they can be determined once for a given machine tool, by performing a series of impulse tests. The variable element in the system is the workpiece. Most often, for RCA applications, the workpiece is modelled analytically as a Timoshenko beam, however, due to a more complex geometry, in the presented example, the workpiece is modelled using the finite element method, as described in Section 2.4. For RCA modal synthesis, the dynamic properties of the components (w index—workpiece, s index—spindle) are noted as matrix equations:

(17) $$\left[\begin{array}{c} x_{s1}\\ \varphi_{s1} \end{array}\right]=\left[\begin{array}{cc} H_{s11} & L_{s11}\\ N_{s11} & P_{s11} \end{array}\right]\cdot \left[\begin{array}{c} F_{s1}\\ M_{s1} \end{array}\right]$$

(18) $$\left[\begin{array}{c} x_{w1}\\ \varphi_{w1}\\ x_{w2} \end{array}\right]=\left[\begin{array}{ccc} H_{w11} & L_{w11} & H_{w12}\\ N_{w11} & P_{w11} & N_{w12}\\ H_{w21} & L_{w21} & H_{w22} \end{array}\right]\cdot \left[\begin{array}{c} F_{w1}\\ M_{w1}\\ F_{w2} \end{array}\right]$$

where: *x*—translational displacement in direction x, *φ*—angle of rotation, *F*—force, *M*—torque, transfer functions: translational *H*[m/N] and rotational *L*[m/Nm], *N*[rad/N], *P*[rad/Nm].

The matrix equation describing the dynamic properties of the combined system shall be noted as:

(19) $$\left[\begin{array}{c} x_{1}\\ \varphi_{1}\\ x_{2} \end{array}\right]=\left[\begin{array}{ccc} H_{11} & L_{11} & H_{12}\\ N_{11} & P_{11} & N_{12}\\ H_{21} & L_{21} & H_{22} \end{array}\right]\cdot \left[\begin{array}{c} F_{1}\\ M_{1}\\ F_{2} \end{array}\right]$$

Having both a model of the dynamic properties of the machine tool and the workpiece, boundary conditions and force balance conditions between the components are noted as follows:

(20) $$\begin{array}{cc} \{\begin{array}{c} x_{s1}=x_{w1}=x_{1}\\ \varphi_{s1}=\varphi_{w1}=\varphi_{1} \end{array}, & \{\begin{array}{c} F_{s1}+F_{w1}=F_{1}\\ M_{s1}+M_{w1}=M_{1} \end{array} \end{array}$$

By making appropriate transformations using Equations (17), (18), (20), it is possible to determine the dynamic properties matrix of the combined system:

(21) $$\left[\begin{array}{ccc} H_{11} & L_{11} & H_{12}\\ N_{11} & P_{11} & N_{12}\\ H_{21} & L_{21} & H_{22} \end{array}\right]=\begin{array}{cc} {\left[\begin{array}{ccc} 1+\frac{H_{w11}P_{s11}-H_{w12}L_{s11}}{H_{s11}P_{s11}-L_{s11}N_{s11}} & \frac{H_{w12}H_{s11}-H_{w11}L_{s11}}{H_{s11}P_{s11}-L_{s11}N_{s11}} & 0\\ \frac{H_{w11}P_{s11}-P_{w11}L_{s11}}{H_{s11}P_{s11}-L_{s11}N_{s11}} & 1+\frac{P_{w11}H_{s11}-N_{w11}L_{s11}}{H_{s11}P_{s11}-L_{s11}N_{s11}} & 0\\ \frac{H_{w21}P_{s11}-L_{w21}L_{s11}}{H_{s11}P_{s11}-L_{s11}N_{s11}} & \frac{L_{w21}H_{s11}-H_{w21}L_{s11}}{H_{s11}P_{s11}-L_{s11}N_{s11}} & 1 \end{array}\right]}^{-1}\cdot & \left[\begin{array}{ccc} H_{w11} & L_{w11} & H_{w12}\\ N_{w11} & P_{w11} & N_{w12}\\ H_{w21} & L_{w21} & H_{w22} \end{array}\right] \end{array}$$

**Figure 14:**
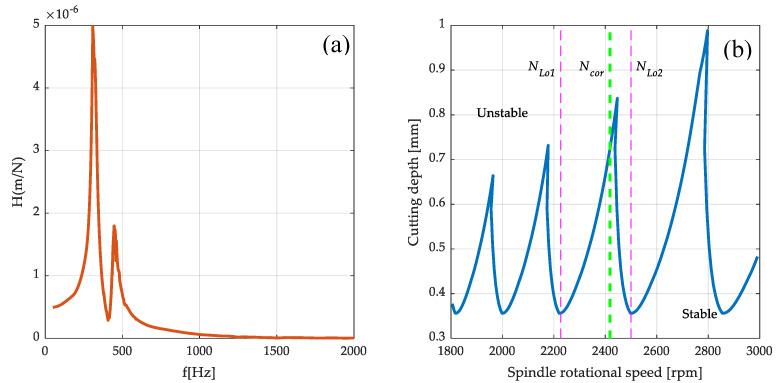
Stability analysis: (**a**) FRF at the end of the workpiece based on RCA (**b**) Stability lobes.

For the stability analysis, the translational function of the transition $H_{22}$ to the x (Figure 14a) direction is used, as it is the highest susceptibility at the end of the workpiece, which is equivalent to the highest risk of vibration occurrence during machining. The tool for predicting system stability is the stability lobe diagram (SLD). The formation of self-excited vibrations during machining is associated with exceeding the cutting depth limit $a_{lim}$, which is presented as follows:

(22) $$a_{lim}=-\frac{1}{2K_{r}Re\left(H\left(j\omega \right)\right)}$$

where Re(H(jω)—the real part of the translational transfer function for a susceptible component of the system, $K_{r}$—coefficient of the cutting forces. As it results from formula (14), positive values of the machining depth limit are obtained for negative values of the function Re(H(jω), which are used to build the SLD diagram. The reference of the machining depth limit to the spindle speed is realized by replicating the lobe with the following relationship:

(23) $$\begin{array}{cc} N_{c}=\frac{60\cdot f_{c}}{k}, & \mathrm{for}\;k=1,2\ldots ,n \end{array}$$

where $f_{c}$—is the chatter frequency, k—consecutive integers denoting the lobe number.

The stability lobes generated in the speed range from 1800 to 3000 rpm for the transfer function under consideration $H_{22}$ are shown in Figure 14a. The stability lobes delineate the areas of technological parameters for which vibrations do not occur (‘Stable’ area) and the area in which self-excited vibrations will develop (‘Unstable’ area).

When analyzing the stability lobes, it should be noted that the selected rotational speeds are more resistant to the occurrence of vibrations during machining. The idea of the presented support for the selection of technological parameters assumes proposing the correction of arbitrarily adopted spindle speeds to the nearest values allowing one to obtain stable machining. The authors experience shows that indicating peaks of the stability lobes as the recommended speeds does not give the expected results due to the proximity of the stability limit. In the proposed approach, first, rotational speeds $N_{Lo1}$ and $N_{Lo2}$ are selected, which define the single lobe range, as shown in Figure 14b. Then, the recommended spindle speed correction $N_{cor}$ for the lobe is determined as:

(24) $$N_{cor}=\left(N_{Lo2}-N_{Lo1}\right)\cdot P_{sh}+N_{Lo1}$$

where $P_{sh}$ is a peak shift coefficient, in the presented system, with the value $P_{sh}=0.7$. The precise determination of the cutting depth is possible with the value of the specific cutting force coefficient determined experimentally for a given material and tool configuration. In the presented system, it was decided to adopt an average value of this coefficient for steel, which significantly simplifies the procedure of supporting the selection of rotational speed, and at the same time does not affect the position of ‘stable’ rotational speeds.

### 2.6. Computer-Aided Manufacturing (CAM)

The technological parameters for which stable conditions and favorable machining performance have been achieved while maintaining the recommended blade life have been imported into the Solidworks CAM system lathe module. Solidworks CAM enables the development of 2.5 and 3 axis turning and milling processes. The software is based on the CAMWorks system. The CAM module implemented in Solidworks provides full support for configuration and parts, which enables the use of geometric features recognized on the basis of the model and the corresponding CAD operations.

Solidworks CAM uses rule-based machining principles, allowing to program the most important machining strategies in the system to be used as standard. These rules can be automatically applied depending on the material type and geometry of the operation.

The system is equipped with an operation recognition function, so it identifies standard geometric primitives such as ruled figures—holes, cylinders. This makes it possible to automate the process of tool path generation by identifying properties from the project tree (feature). For rotating parts, these features are even expected to be optimal. Most importantly, in the application to the research work described in this paper, Solidworks CAM is fully parametrically integrated into the Solidworks system’s graphical kernel, so all modifications to parts are taken into account when rebuilding tool paths and post-processing.

## 3. Results

The first step in verifying the effectiveness of the proposed approach was to carry out the machining of the part without any additional support system for the selection of technological parameters. The machining program was generated in the CAM system, and the technological parameters were selected arbitrarily, taking into account the catalogue data provided by the tool manufacturer and the experience of the technologist.

**Figure 15:**
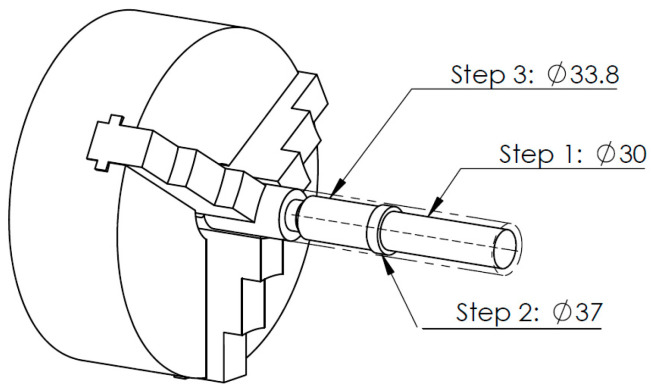
Orientation of the parts to be machined in relation to the prefabricated item.

The assumption was that the object was to be made on a CNC lathe in one clamping. The prefabricated product used for processing was a bar made of steel 1.0715 (11SMn30), with a circular cross-section of a diameter of D=40 mm and overhang L=200 mm. The Figure 15 shows the orientation of the part in relation to the prefabricated product.

For shaping, we used SCLCL/R 2020K09P tools equipped with Sandvik CCMT 09T304 PF 4325 plates (Sandvik Coromant, Sandviken, Sweden). Machining was performed on an AFM TAE-35N CNC lathe Andrychowska Fabryka Maszyn DEFUM, Andrychów, Poland), equipped with a Fanuc control system (Fanuc Robotics Ltd., Oshino, Japan).

**Figure 16:**
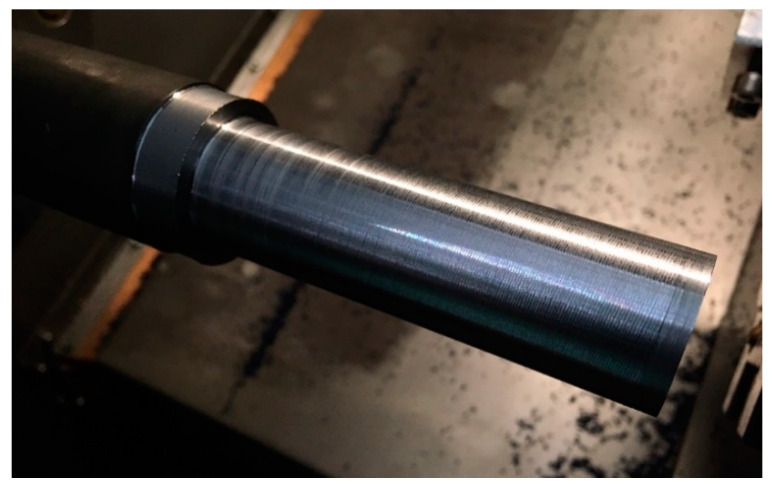
Surface after machining without the system supporting the selection of technological parameters.

Due to the geometry of the machined part in Steps 1 and 2 (Figure 15), the left-hand tool is used for machining. The highest susceptibility and therefore the highest risk of vibration occurs in Step 1, for which the diameter of the workpiece is to be 30 mm. The machining is carried out at a constant machining speed $v_{c}=190\;m/\min$, depth of cut $a_{p}=1.0\;\mathrm{mm}$ and feed rate $f_{n}=0.1\;\mathrm{mm}/rev$. The surface after machining for Steps 1 and 2 is shown in Figure 16.

The surface machined in Step 1 shows the characteristic trace of vibration occurrence during the machining. When machining the last layer, on the diameter of D=30 mm for the selected machining speed, the spindle speed was N=2016 rpm. When analyzing the course of the stability lobes shown in Figure 14b, it should be noted that there is an increased risk of vibrations during machining at a given rotational speed.

**Figure 17:**
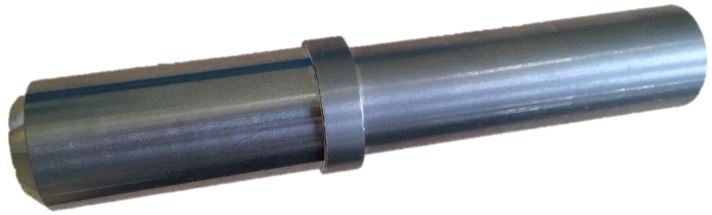
The part after machining with the support of technological parameters selection.

The next step was to make a part, taking into account the conducted process stability analysis. The application of the module supporting the selection of machining parameters allowed one to introduce a correction of the spindle speed to the nearest ‘stable’ speeds, in order to minimize the risk of vibration during machining. In the selected case, the corrected spindle speed was $N_{cor}=2154\;\mathrm{rpm}$. Figure 17 presents the finished part with the rotational speeds proposed by the developed support system. No vibrations were observed during the machining, which is confirmed by the obtained surface condition.

**Figure 18:**
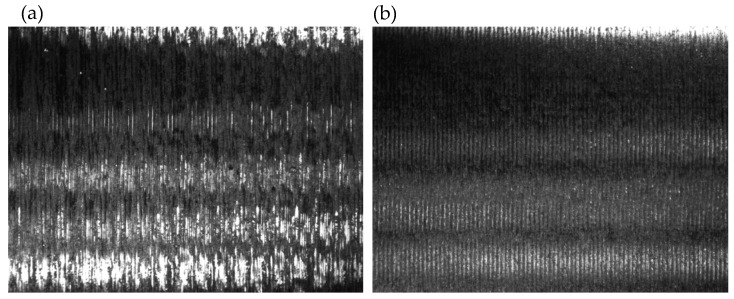
Surface comparison in Step 1: (**a**) without technological parameters selection support (**b**) with the support.

Figure 18 presents a comparison of the surface condition in Step 1 for the part manufactured without analysis of the stability of the cutting process, (a) and for the part where the proposed system of technological parameters support was used (b). The photographs showing the machined surfaces were taken using the Hawk Elite measuring microscope (Vision Engineering Ltd., Woking, UK) with a 20× magnification lens.

The comparison shows a clear improvement in surface condition due to the rotational speed correction. No signs of vibration were observed on surface (b), while these are clearly visible on surface (a). It is particularly important to note that the observed improvement was achieved using a software solution only, by changing the set rotational speed.

**Figure 19:**
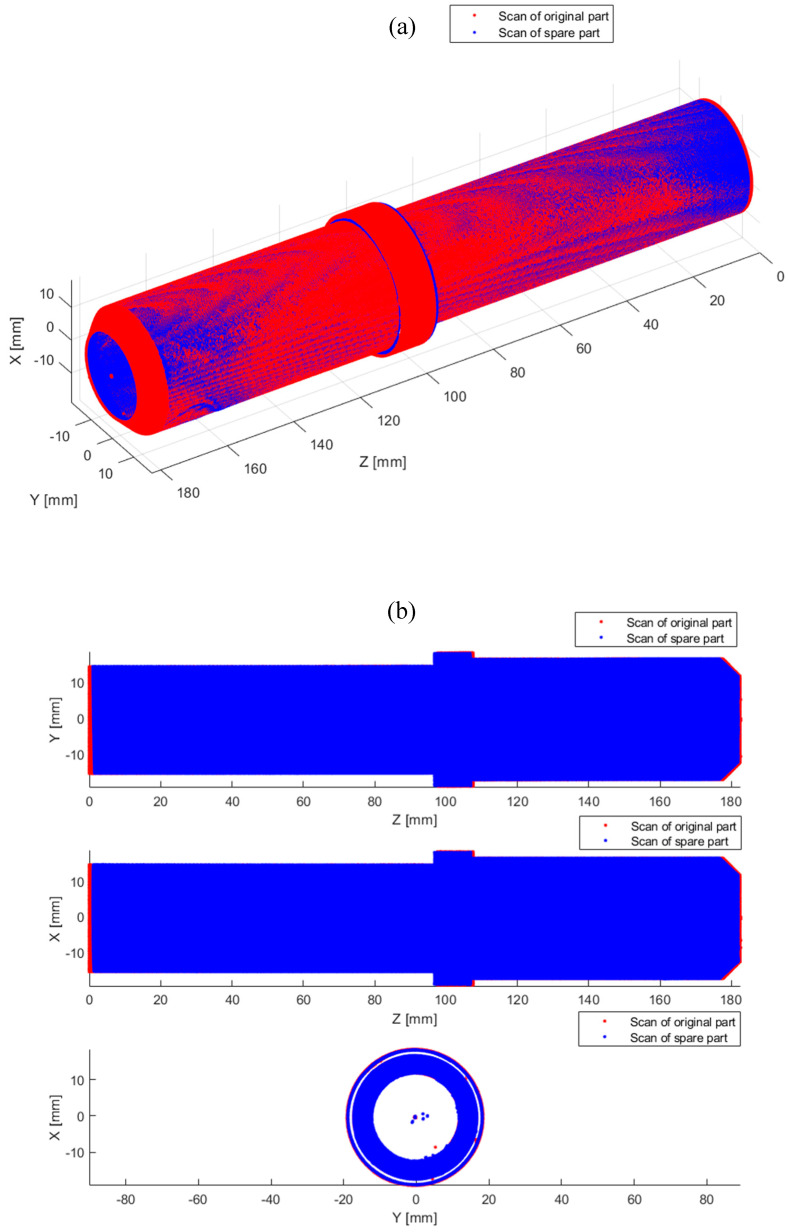
Comparison of 3D scans of the original part and a manufactured spare part, (**a**) 3D view (top), (**b**) side view (bottom).

The last step of the verification was a 3D scan of the machined spare part and a comparison with the geometry of the original part. Overlapping geometries are shown in Figure 19.

In Figure 19, the blue color indicates the original part, while the red color indicates the scan of the spare part produced using the proposed system. The resulting discrepancies expressed in the form of RMSE are 0.15 mm, which corresponds to the accuracy of the scanner used.

## 4. Conclusions

The paper presents the concept of a system, allowing one to simplify the procedure of manufacturing spare parts. The presented concept allows one to manufacture a spare part on the basis of the original part scan. Such an approach is particularly useful when manufacturing spare parts for special machines, where access to technical documentation or finished parts is difficult or impossible.

The feature recognition module presented is characterized by the high accuracy of cylindrical elements and chamfers recognition. The result of this module is a parametric CAD model. On the basis of the obtained model, technological strategies and parameters of the processing are determined to minimize the risk of vibration occurrence during processing. Such an approach enables obtaining the required dimensional and shape accuracy and high quality of the machined surface.

The main limitation of the presented system is the heterogeneity of computing environments. Therefore, the direction of system development is its unification. Moreover, it is planned to extend the functionality of the system by recognizing further geometric features (e.g., roundness, conical surfaces, cavities) and to introduce the possibility of generating a CAD model based on damaged surfaces.

To sum up, the presented system allows for the highly automated production of copies of special machine parts, thus reducing the need to engage qualified staff and specialized measuring equipment, while providing technological processing parameters beneficial from the point of view of process stability.

## Figures and Tables

**Table 1:** Matrix of recognized geometric properties.

No.	X	Y	R	Z Start	Z End	Chamfer Start	Chamfer End
1	0	0	15	0	98	0.0	0.0
2	0	0	18.5	98	108	0.0	0.0
3	0	0	16.9	108	183	0.0	5.0

**Table 2:** Pseudocode of geometry import algorithm to SOLIDWORKS software.

**Input: Matrix of recognized geometric properties** **Output: Parametric CAD model**
1. Determine the number of steps (based on Matrix of properties)
2. Create ‘New Part’
3. Open Sketch on XZ plane
4. Draw contour of the basic geometry
5. Create the basic geometry using ‘Revolved Boss/Base’ function
6. Find edges to chamfer
7. For i = number of edges to chamfer
8. Specify the type of chamfer 9. Create sketch of chamfer on XZ plane
10. Create chamfer using ‘Recolved Boss/Base’ or ‘Revolved Cut’ (type of function dependent on the specified type of chamfer)
11. Save CAD model in specified format
